# Supplementary material for: Food Parenting Practices among Parents with Overweight and Obesity: A Systematic Review
Source: Nutrients. 2018 Dec 12;10(12):1966. doi: 10.3390/nu10121966 (PMC6316864; doi:10.3390/nu10121966)
Supplement: Supplementary file 1 [file nutrients-10-01966-s001.zip › supplementary-final check/S1 Database search strategies.docx]

**Supplementary File 1**

Example search strategies

Web of Science

(TS=((feeding or mealtime*) AND child* AND obes* AND (parent* or mother* or father* or maternal or paternal))) AND LANGUAGE: (English) AND DOCUMENT TYPES: (Article)

PubMed

(((((((((parent*[Title/Abstract]) OR mother*[Title/Abstract]) OR father*[Title/Abstract]) OR caregiver*[Title/Abstract]) OR maternal[Title/Abstract]) OR paternal[Title/Abstract])) AND (((((((chlid*[Title/Abstract]) OR toddler*[Title/Abstract]) OR adolescen*[Title/Abstract]) OR daughter*[Title/Abstract]) OR son*[Title/Abstract]) OR girl[Title/Abstract]) OR boy[Title/Abstract])) AND (((((feeding practice*[Title/Abstract]) OR feeding behav*[Title/Abstract]) OR feeding pattern*[Title/Abstract]) OR feeding[Title/Abstract]) NOT breastfeeding[Title/Abstract])) AND ((((obes*[Title/Abstract]) OR overweight[Title/Abstract]) OR BMI[Title/Abstract]) OR body mass index[Title/Abstract])

PsycINFO

((((feeding OR mealtime*) AND child* AND obes* AND (parent* OR mother* OR father* OR maternal OR paternal)) AND (la.exact("ENG") NOT me.exact("Systematic Review" OR "Meta Analysis") NOT po.exact("Animal") NOT rtype.exact("Comment/Reply" OR "Editorial" OR "Erratum/Correction" OR "Review-Book" OR "Column/Opinion" OR "Letter") AND PEER(yes))) NOT (me.exact("Prospective Study" OR "Clinical Trial" OR "Mathematical Model" OR "Twin Study" OR "Brain Imaging") NOT po.exact("Animal") NOT rtype.exact("Review-Book" OR "Column/Opinion" OR "Letter") AND PEER(yes))) NOT (fdb(10000250 10000244 10000233 10000243 1007025 1007418 10000254 1005684 10000003 1007428 1007426 1007551 1007156 1007566 1007455 1007427 1007489 1007522 1007422 1007529) AND at.exact("Article" NOT ("Review" OR "News" OR "Literature Review" OR "Undefined" OR "Commentary" OR "Conference" OR "Editorial")) AND stype.exact("Scholarly Journals") AND la.exact("ENG") NOT subt.exact("metabolism" OR "breast feeding" OR "diabetes" OR "breastfeeding & lactation" OR "rodents" OR "diabetes mellitus" OR "insulin" OR "birth weight" OR "fatty acids" OR "proteins" OR "gene expression" OR "systematic review" OR "neonates" OR "homeostasis" OR "microbiota" OR "fetuses" OR "glucose" OR "data processing" OR "intestine" OR "insulin resistance" OR "birth" OR "liver") AND pd(20170101-20181231) AND PEER(yes))
